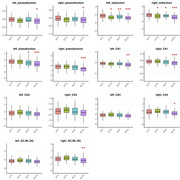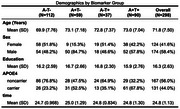# Biomarker‐Driven Patterns of Hippocampal Subfield Atrophy in Amnestic Mild Cognitive Impairment

**DOI:** 10.1002/alz70856_107740

**Published:** 2026-01-09

**Authors:** Andrei Bieger, Wyllians Vendramini Borelli, João Pedro Ferrari‐Souza, Wagner S. Brum, Thomas Hugentobler Schlickmann, Daniel Arnold, Tharick A Pascoal, Diogo O. Souza, Cristiano Aguzzoli, Pedro Rosa‐Neto, Lucas Porcello Schilling, Eduardo R. Zimmer, Alzheimer's Disease Neuroimaging Initiative (ADNI)

**Affiliations:** ^1^ Universidade Federal do Rio Grande do Sul, Porto Alegre, Rio Grande do Sul, Brazil; ^2^ Universidade Federal do Rio Grande do Sul, Porto Alegre, RS, Brazil; ^3^ University of Pittsburgh, Pittsburgh, PA, USA; ^4^ UFRGS, Porto Alegre, Rio Grande do Sul, Brazil; ^5^ Instituto do Cérebro do Rio Grande do Sul, Porto Alegre, Rio Grande do Sul, Brazil; ^6^ Montreal Neurological Institute, Montreal, QC, Canada; ^7^ Brain Institute of Rio Grande do Sul (InsCer), Porto Alegre, Rio Grande do Sul, Brazil; ^8^ Laboratory of Neuro Imaging (LONI), University of Southern California, Los Angeles, CA, USA

## Abstract

**Background:**

Hippocampal atrophy is a well‐established hallmark of Alzheimer's disease (AD), closely associated with cognitive decline and disease progression, even in the earliest symptomatic stages. Despite growing evidence that subfield‐specific atrophy patterns could serve as sensitive biomarkers for early diagnosis and tracking disease progression, there remains a significant gap in understanding how these subfields are differentially impacted across different stages of AD. In this study, we explore longitudinal rates of atrophy in hippocampal subfields in individuals with amnestic mild cognitive impairment (MCI).

**Method:**

Hippocampal subfields volumes were extracted from volumetric, T1‐weighted MRI images from the Alzheimer's Disease Neuroimaging Initiative using Freesurfer (v 7.4.1). We included individuals with MCI who underwent cerebrospinal fluid (CSF) collection and MRI studies at baseline and after 1 year. Participants were categorized according to baseline CSF levels of amyloid and phosphorylated‐tau proteins as: A‐T‐, A+T‐, A‐T+, and A+T+. Using generalized linear models, we evaluated the effect of biomarker subgroups on hippocampal subfield atrophy rates, controlling for age, sex, and whole hippocampal volume.

**Result:**

Table 1 summarizes demographic data for each biomarker group. Using the A‐T‐ group as reference, higher rates of atrophy were observed in the A‐T+ group in the subiculum, bilaterally. The A+T‐ group showed differences in the left presubiculum and in the subiculum, bilaterally. In the A+T+ group, faster atrophy was observed in the bilateral parasubiculum, bilateral subiculum, bilateral presubiculum, bilateral CA1, right CA4, and right dentate gyrus (granule cell and molecular layers) ‐ Figure 1.

**Conclusion:**

This study reveals distinct patterns of hippocampal subfield atrophy in amnestic MCI, according to amyloid‐beta (A) and phosphorylated tau (T) biomarker profiles. The A‐T+ group exhibited a unique pattern of subfield vulnerability, primarily affecting the subiculum, suggesting that tau pathology alone may target specific regions of the hippocampus. In contrast, the A+T+ group showed widespread atrophy across multiple subfields, indicating that combined amyloid and tau pathology leads to more extensive hippocampal degeneration. These findings highlight the potential of subfield‐specific atrophy patterns as sensitive biomarkers for differentiating early AD stages and tracking disease progression. Future studies should investigate how these patterns correlate with cognitive decline and their predictive value for dementia conversion.